# Microbial Ecology of Thailand Tsunami and Non-Tsunami Affected Terrestrials

**DOI:** 10.1371/journal.pone.0094236

**Published:** 2014-04-07

**Authors:** Naraporn Somboonna, Alisa Wilantho, Kruawun Jankaew, Anunchai Assawamakin, Duangjai Sangsrakru, Sithichoke Tangphatsornruang, Sissades Tongsima

**Affiliations:** 1 Department of Microbiology, Faculty of Science, Chulalongkorn University, Bangkok, Thailand; 2 Genome Institute, National Center for Genetic Engineering and Biotechnology, Pathumthani, Thailand; 3 Department of Geology, Faculty of Science, Chulalongkorn University, Bangkok, Thailand; 4 Department of Pharmacology, Faculty of Pharmacy, Mahidol University, Bangkok, Thailand; Laurentian University, Canada

## Abstract

The effects of tsunamis on microbial ecologies have been ill-defined, especially in Phang Nga province, Thailand. This ecosystem was catastrophically impacted by the 2004 Indian Ocean tsunami as well as the 600 year-old tsunami in Phra Thong island, Phang Nga province. No study has been conducted to elucidate their effects on microbial ecology. This study represents the first to elucidate their effects on microbial ecology. We utilized metagenomics with 16S and 18S rDNA-barcoded pyrosequencing to obtain prokaryotic and eukaryotic profiles for this terrestrial site, tsunami affected (S_1_), as well as a parallel unaffected terrestrial site, non-tsunami affected (S_2_). S_1_ demonstrated unique microbial community patterns than S_2_. The dendrogram constructed using the prokaryotic profiles supported the unique S_1_ microbial communities. S_1_ contained more proportions of archaea and bacteria domains, specifically species belonging to Bacteroidetes became more frequent, in replacing of the other typical floras like Proteobacteria, Acidobacteria and Basidiomycota. Pathogenic microbes, including *Acinetobacter haemolyticus*, *Flavobacterium* spp. and *Photobacterium* spp., were also found frequently in S_1_. Furthermore, different metabolic potentials highlighted this microbial community change could impact the functional ecology of the site. Moreover, the habitat prediction based on percent of species indicators for marine, brackish, freshwater and terrestrial niches pointed the S_1_ to largely comprise marine habitat indicating-species.

## Introduction

**Figure 1:**
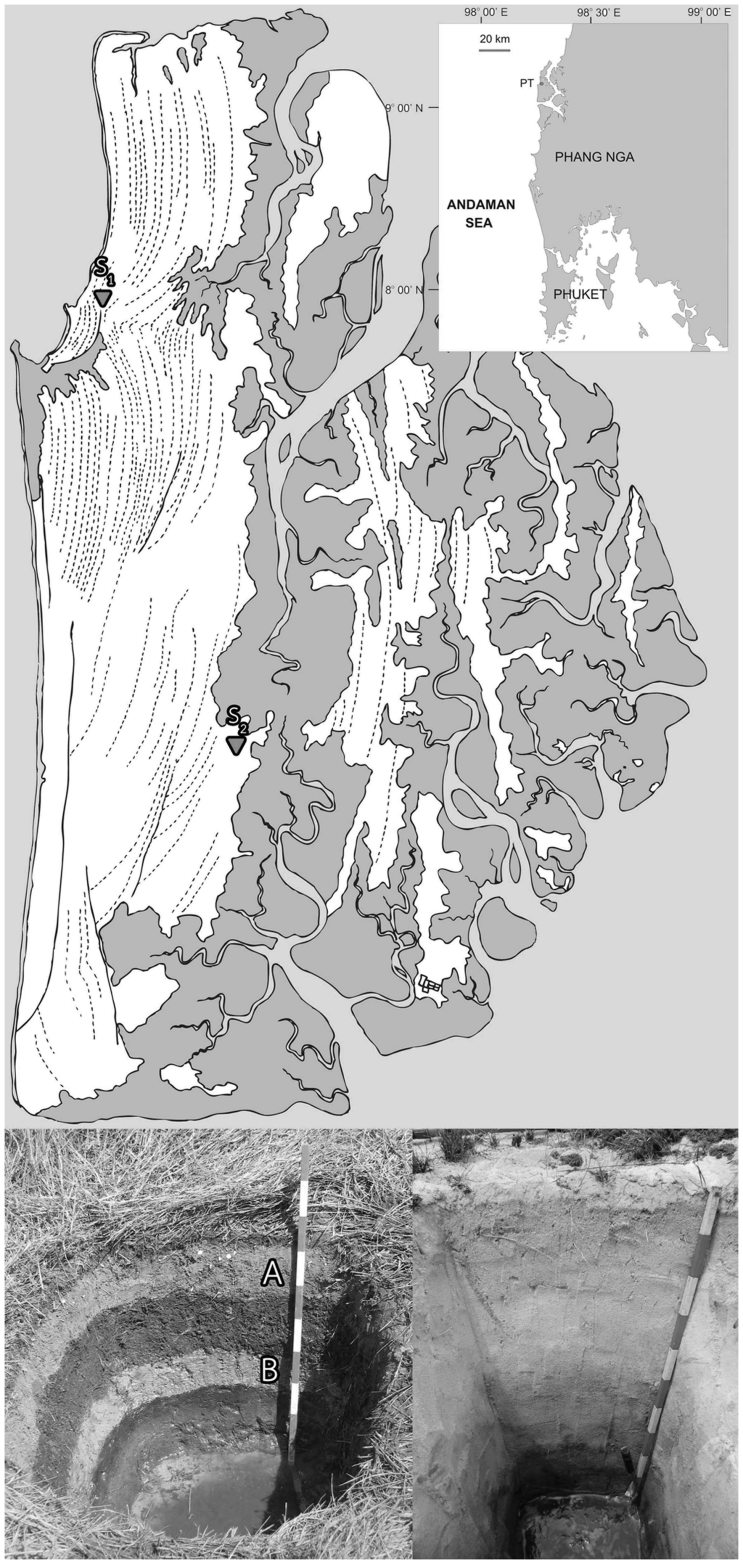
Index map of Phra Thong island relative to Phuket and terrestrial sites where samples were collected. The lower left photograph shows the pit wall of tsunami affected site (S_1_). Light color sheets **A** and **B** represent 2004 tsunami and 600yo tsunami affected terrestrial layers, respectively [1]. The lower right photograph shows the pit wall of non-tsunami affected site (S_2_) of the parallel geolography, and samples of equivalent depths to those of S_1_ were collected. Time period of the terrestrial is determined via sample depth [1].

Phra Thong island, Phang Nga province of southern Thailand (Figure 1), represents a location for comparative studies of tsunami (S_1_) and non-tsunami (S_2_) affected terrestrial ecosystems. The S_1_and S_2_ shared nearby geographies separated by a hill, whereby S_1_ terrain was inundated by the Indian Ocean tsunami on 26 December 2004 and S_2_ unaffected; otherwise both were comparable based on geological characteristics [1], [2]. The tsunami left an Andaman Sea-facing, S_1_, distinguished terrestrial layer that was classified by geologist as a sand layer of 5–20 cm thick (layer A in Figure 1; [1]). Interestingly, geological evidence indicated three historic tsunamis also occurred prior to the 2004 tsunami at S_1_, and none to S_2_. The youngest recorded historic tsunami predating the 2004 tsunami was approximately 600 years ago (600yo) (layer B in Figure 1; [1]).

Each tsunami occurrence could affect the S_1_ terrestrial characteristics due to the massive impact of seawater with marine organisms and garbage [1], [3], [4]. Studies comparing the 2004 tsunami affected versus non-affected (or pre-affected) terrestrials and terrestrial water reported the greater salinity, acidity, conductivity, turbidity and organic contents following the tsunami occurrence [5]–[7]. Studies also reported widespread disease-carrying vectors, such as mosquitoes, trematodes and snails, after the 2004 tsunami [3], [4]. Several bacterial and fungal infections involved skin and respiratory disorders were documented among repatriated tourists [8] and people working in the tsunami affected area [9]. In addition, the 2004 tsunami sediments consisted of higher concentrations of Mercury and Thallium [10], [11]. Together, this chance of terrestrial characteristics could affect the microbial biodiversity and functional ecology.

Nonetheless, the impact of tsunamis on microbial diversity and ecology function remains ill-defined. The present study thereby analyzed the microbial biodiversity and their potential functional composites in the tsunami impacted S_1_ terrain, in comparison to the non-affected S_2_ site, using 16S and 18S rRNA genes pyrosequencing derived metagenomic DNA approach. For each site, the data included the prokaryotic and eukaryotic diversity profiles categorized into different depth levels corresponding to the terrestrial ages: 2004 tsunami, 1–300yo (pre-dating the 2004), 300–600yo, 600yo tsunami, and >600yo, respectively (starting from the top layer to a deeper layer), and also the amalgamated profiles for each site. Geologists determined the terrestrial age period from its depths below the land surface [1]. The overall results represent for the first time the use of metagenomics in analysing the prokaryotic and eukaryotic microbial biodiversity of the 2004 tsunami and non-tsunami affected terrestrials. Unlike traditional biodiversity study that is conducted via cultivation technique and could reveal merely less than 1% of the true microbiota, metagenomics is a culture-independent technique that has been proved worldwide a robust, reliable and comprehensive tool for obtainment of entire microbiota from diverse environmental and clinical samples [12]–[17].

## Materials and Methods

### Sample collection

The owners of the lands gave permission to conduct the study on these sites. We confirm that the study did not involve endangered or protected species.

Phra Thong island provides a location for comparative tsunami (S_1_: N9.13194 E98.26250) and non-tsunami (S_2_: N9.07250 E98.27222) affected terrestrial studies based on geological evidences (Jankaew, personal communication) [1], [2]. S_1_ and S_2_ are 6.73 km apart. S_1_ is 0.40 km from the sea, and S_2_ is 2.26 km from the sea (Figure 1). Approximately 1 kg samples were collected, each in sterile containers, between 11:00–15:00 hours during 23–24 March 2011. S_1_ samples comprised: 2004 tsunami (14.5 cm), 1–300yo (22 cm), 300–600yo (29 cm), 600yo tsunami (38 cm), and >600yo (46 cm); S_2_ samples comprised: S21 (14.5 cm), S22 (22 cm), S23 (29 cm), S24 (38 cm), and S25 (46 cm). The number in parenthesis represents the depth level where the sample was collected, and that entailed the approximate age of the sample relative to the year 2004. On-site records for color, texture and pH were done. All samples were transported in ice chest, stored in 4°C and processed for the next steps within 14 days.

### Metagenomic DNA extraction and DNA quality examination

Each sample was mixed with a sterile spatula, and 15 g each was used for metagenomic DNA extraction [18]. Two independent metagenomic DNA extractions were performed per sample. The samples were dissolved in an extraction buffer (Epicentre, Wisconsin, USA) with Tween 20, low-speed centrifuged to remove large debris, and poured through four-layered sterile cheesecloth to remove particles and organisms of >30 μm in size. Microorganisms between 0.22 and 30 μm were collected by filtering over a sterile 0.22 μm filter membrane (Merck Millipore, Massachusetts, USA) [15]. Total nucleic acid from each sample was extracted using Meta-G-Nome DNA Isolation Kit (Epicentre) following the manufacturer's protocols. Metagenomic DNA quality was assessed using agarose gel electrophoresis. The DNA concentration and purity was further analysed by A_260_ and the ratio of A_260_/A_280_ spectrophotometry, respectively.

### PCR generation of pyrotagged 16S and 18S rDNA libraries

Table 1 lists forward and reverse pyrotagged 16S and 18S rRNA gene primers. For broad-range 16S and 18S rRNA genes amplification, universal prokaryotic 338F (forward) and 803R (reverse) primers [19]–[21], and universal eukaryotic 1A (forward) and 516R (reverse) primers [15], [22], [23] were used. Italics denote the eight nucleotides pyrotag sequences, functioning to specify sample names [24]. A 50-μl PCR reaction comprised 1× EmeraldAmp GT PCR Master Mix (TaKaRa, Shiga, Japan), 0.3 μM of each primer, and 100 ng of the metagenome. PCR conditions were 95°C for 4 min, and 30–35 cycles of 94°C for 45 s, 50°C for 55 s and 72°C for 1 min 30 s, followed by 72°C for 10 min. To generate the pyrotagged 16S or 18S rDNA libraries with minimized stochastic PCR biases, two to three independent PCRs were performed per extracted metagenomes, and two extracted metagenomes per sample, resulting in a minimum of four PCR products to be pooled for pyrosequencing per sample.

**Table 1:** Pyrotagged16S and 18S rRNA genesuniversal primers.

Sample names	Forward primers (5′-3′)	Reverse primers (5′-3′)
**16S rRNA gene universal primers**		
2004 tsunami	*TAGTAGCG* ACTCCTACGGGAGGCAGCAG	*TAGTAGCG* CTACCAGGGTATCTAATC
1-300yo	*AGACGACG* ACTCCTACGGGAGGCAGCAG	*AGACGACG* CTACCAGGGTATCTAATC
300–600yo	*ACTCGTAG* ACTCCTACGGGAGGCAGCAG	*ACTCGTAG* CTACCAGGGTATCTAATC
600yo tsunami	*ACATCGAG* ACTCCTACGGGAGGCAGCAG	*ACATCGAG* CTACCAGGGTATCTAATC
>600yo	*ACGCTATC* ACTCCTACGGGAGGCAGCAG	*ACGCTATC* CTACCAGGGTATCTAATC
S21	*TACTACGC* ACTCCTACGGGAGGCAGCAG	*TACTACGC* CTACCAGGGTATCTAATC
S22	*AGCAGAGC* ACTCCTACGGGAGGCAGCAG	*AGCAGAGC* CTACCAGGGTATCTAATC
S23	*TCAGCTAC* ACTCCTACGGGAGGCAGCAG	*TCAGCTAC* CTACCAGGGTATCTAATC
S24	*AGAGCGAC* ACTCCTACGGGAGGCAGCAG	*AGAGCGAC* CTACCAGGGTATCTAATC
S25	*ATGCTCAC* ACTCCTACGGGAGGCAGCAG	*ATGCTCAC* CTACCAGGGTATCTAATC
**18S rRNA gene universal primers**		
2004 tsunami	*AGATAGCG* CTGGTTGATCCTGCCAGT	*AGATAGCG* ACCAGACTTGCCCTCC
1–300yo	*TGTAGACG* CTGGTTGATCCTGCCAGT	*TGTAGACG* ACCAGACTTGCCCTCC
300–600yo	*TGCAGTAG* CTGGTTGATCCTGCCAGT	*TGCAGTAG* ACCAGACTTGCCCTCC
600yo tsunami	*TCTGCGAG* CTGGTTGATCCTGCCAGT	*TCTGCGAG* ACCAGACTTGCCCTCC
>600yo	*ATCAGCAG* CTGGTTGATCCTGCCAGT	*ATCAGCAG* ACCAGACTTGCCCTCC
S21	*ATACAGTC* CTGGTTGATCCTGCCAGT	*ATACAGTC* ACCAGACTTGCCCTCC
S22	*ATCATATC* CTGGTTGATCCTGCCAGT	*ATCATATC* ACCAGACTTGCCCTCC
S23	*TGCGATGC* CTGGTTGATCCTGCCAGT	*TGCGATGC* ACCAGACTTGCCCTCC
S24	*ATCGCAGC* CTGGTTGATCCTGCCAGT	*ATCGCAGC* ACCAGACTTGCCCTCC
S25	*TATACTAC* CTGGTTGATCCTGCCAGT	*TATACTAC* ACCAGACTTGCCCTCC

Italic sequence denotes the 8 nt-pyrotagged sequence.

### Gel purification and pyrosequencing

PCR products (∼473 bp for 16S rDNAs; ∼577 bp 18S rDNAs) were excised from agarose gels, and purified using PureLink Quick Gel Extraction Kit (Invitrogen, New York, USA). The 454-sequencing adaptors were ligated to all 16S and 18S rDNA fragments, the reactions were purified by MinElute PCR Purification Kit (Qiagen), and the samples were pooled for pyrosequencing on an eight-lane Roche picotiter plate, 454 GS FLX system (Roche, Branford, CT) at the in-house facility of the National Center for Genetic Engineering and Biotechnology, according to the recommendations of the supplier.

### Sequence annotation and bioinformatic analyses

After removal of unreliable sequences, including sequences that failed the pyrosequencing quality cut-off and sequences shorter than 50 nucleotides, the sequences were categorized based on the appended pyrotag sequences. Sequences corresponding to the same sample category were inspected for domain and taxon compositions using mg-RAST [25], [26] with default parameters. Species were identified by BLASTN [27] with E-value ≤10^−5^ against 16S rDNA databases including NCBI non-redundant [28], RDP [29] and Greengenes [30], and for 18S rDNAs the databases included NCBI non-redundant [28], EMBL [31], [32] and SILVA [33]. Evolutionary distances and phylogenetic tree were computed with default thresholds (E-value ≤10^−8^, similarity score ≥80%). Species (or phylum) prevalence was determined by dividing the frequency of reads in the species (or phylum) by the total number of the identifiable reads. The differences in community structures were compared using Yue & Clayton theta similarity coefficients (Thetayc) and Morisita-Horn dissimilarity index, in mothur [34]–[36]. Low Thetayc and Morista-Horn inferred high community similarity. An unweighted pair group method with arithmetic mean (UPGMA) clustering was constructed using Thetayc, in mothur [36]. Furthermore, functional subsystems and functional groups of the prokaryotic profiles were determined using SEED-based assignments in mg-RAST server [25], [26], [37]. For habitat classification, the data were compared against a World Register of Marine Species (WoRMS) database [38].

## Results

### Metagenome abundances and compositions at domain and kingdom levels

On-site records for physical characteristics of S_1_ and S_2_ were as shown in Figure 1. Alternating layers of black soil-like and grey sand-like comprised most of S_1_, whereby more homogeneous layers of grey sand-like predominated in S_2_. Differences in pH were also evident between the two ecosystems where S_1_ ranged from 6–7 (more acidity), while S_2_ ranged from 7–7.5.

Following total nucleic acids extraction of 0.22–30 μm sizes, S_1_ and S_2_ had average metagenomic concentration of 23.16 ng and 27.02 ng per gram of soil, respectively. Libraries of pyrotagged 16S and 18S rRNA gene fragments were constructed, and pyrosequenced to obtain the culture-independent prokaryotic and eukaryotic profiles of the sites. After removal of unreliable sequences, 21,592reads for S_1_ and 33,308 reads for S_2_ remained for BLASTN species identification. Significant E-values (≤10^−5^) were identified for 20,555 reads for S_1_ (95.20%) and 31,946 reads for S_2_ (95.91%). Reads with non-significant E-values (>10^−5^) were omitted from the analyses.

**Figure 2:**
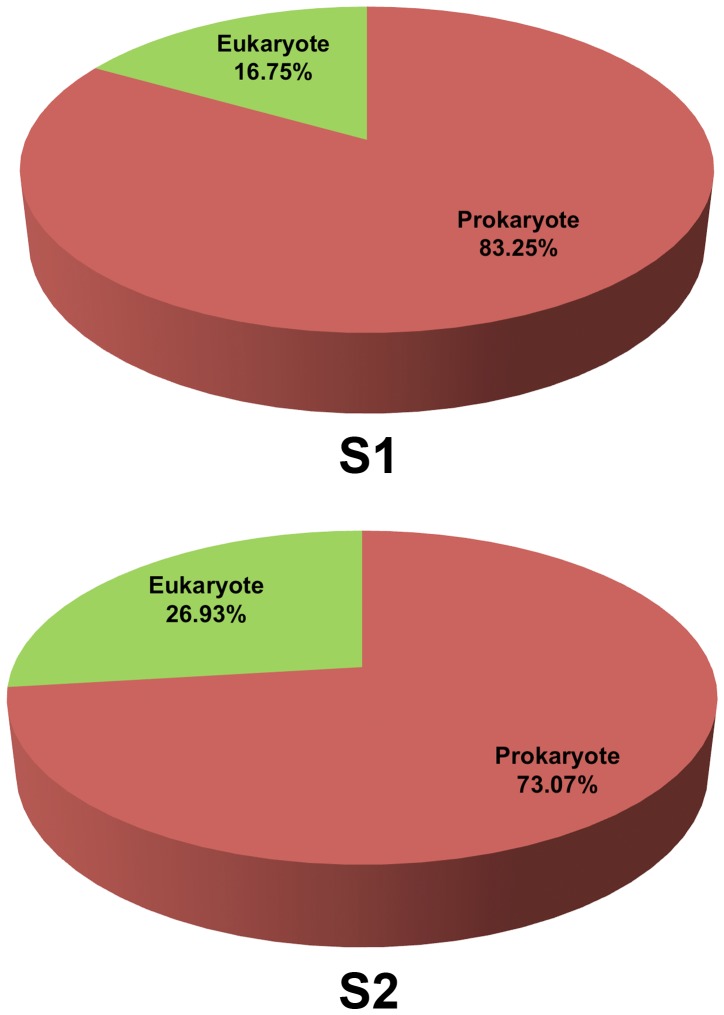
Percentages of prokaryotic and eukaryotic domains in S_1_ and S_2_.

**Figure 3:**
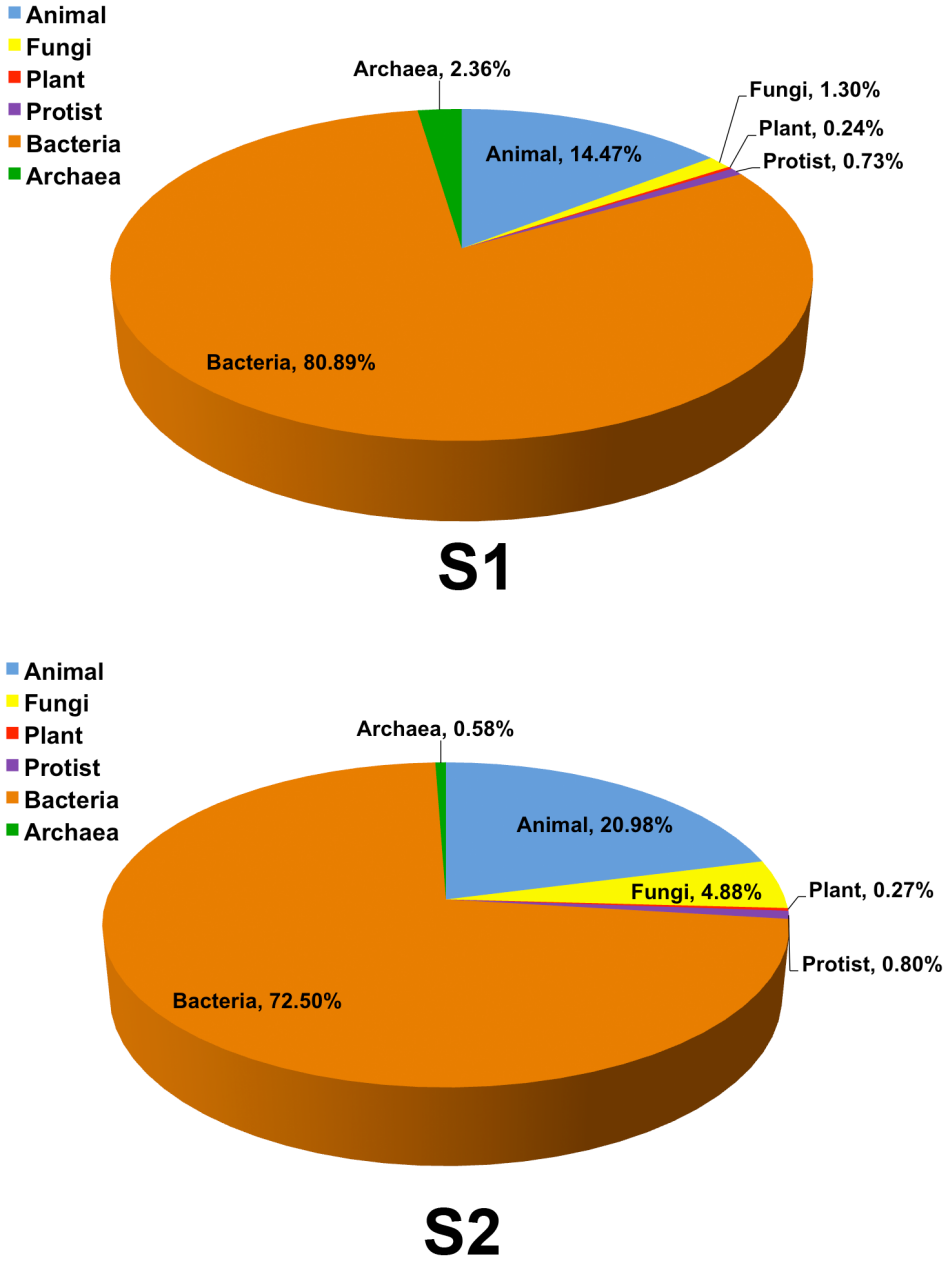
Percentages of 6 kingdoms of lives in S_1_ and S_2_.

The domain compositions of S_1_ indicated a lower proportion of eukaryotes and higher proportion of prokaryotes than S_2_ (Figure 2). The proportion of eukaryotic species was reduced by almost half in S_1_. Among prokaryotes, the greatest divergence between S_1_ and S_2_ was evident among archaea compared to bacteria where found increased in S_1_ (Figure 3: 1.15-fold for bacteria, 4.07-fold archaea). These greater representations somewhat caused the reduced diversity of the other 4 kingdoms of lives.

### Diversity of prokaryotic phyla and species

**Figure 4:**
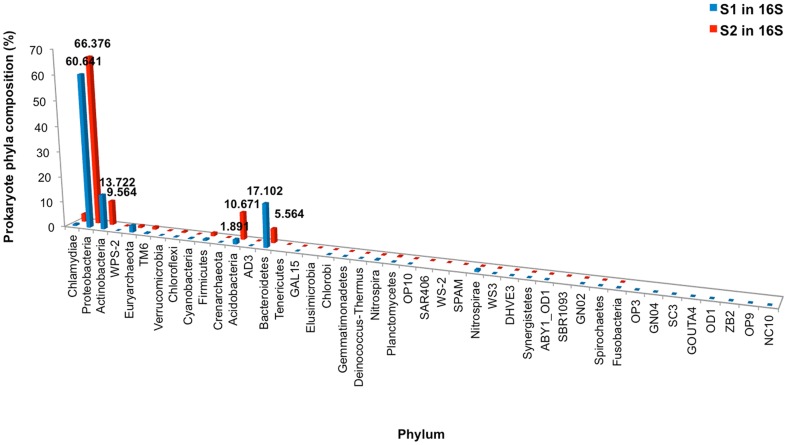
Distribution of prokaryotic phyla in S_1_ and S_2_, without (A) and with (B) individual sample ages categorization.

**Figure 5:**
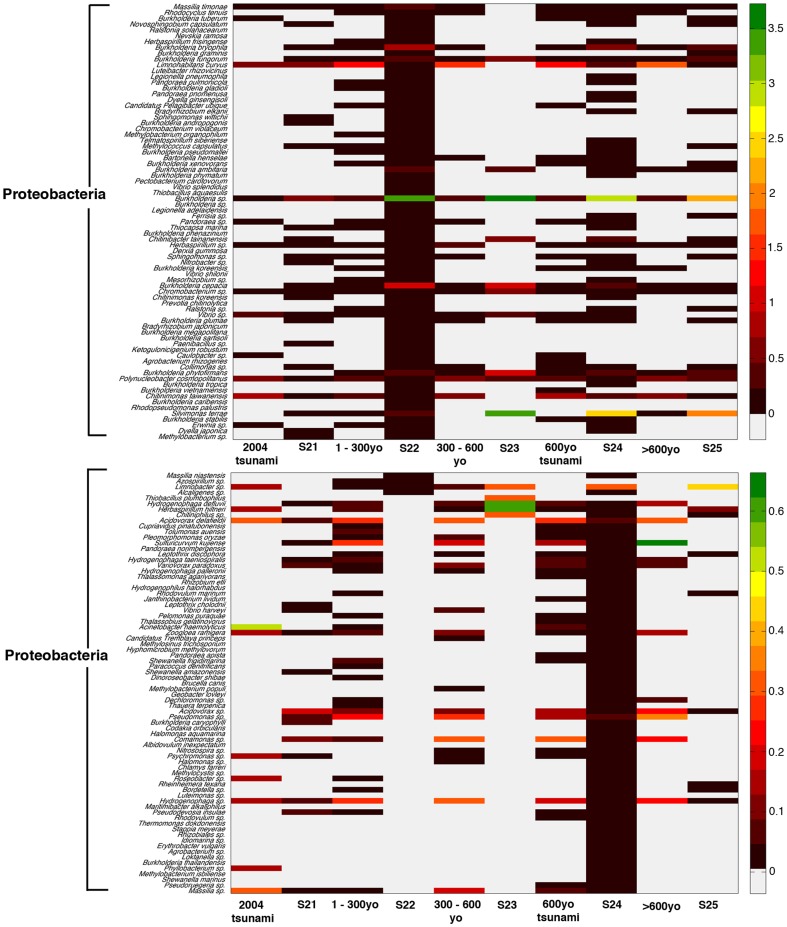
Distribution of prokaryotic species in S_1_ and S_2_, categorized by individual sample ages. Different color on the diagram represents a different relative abundance, based on the percent frequency chart on the right.

**Figure 6:**
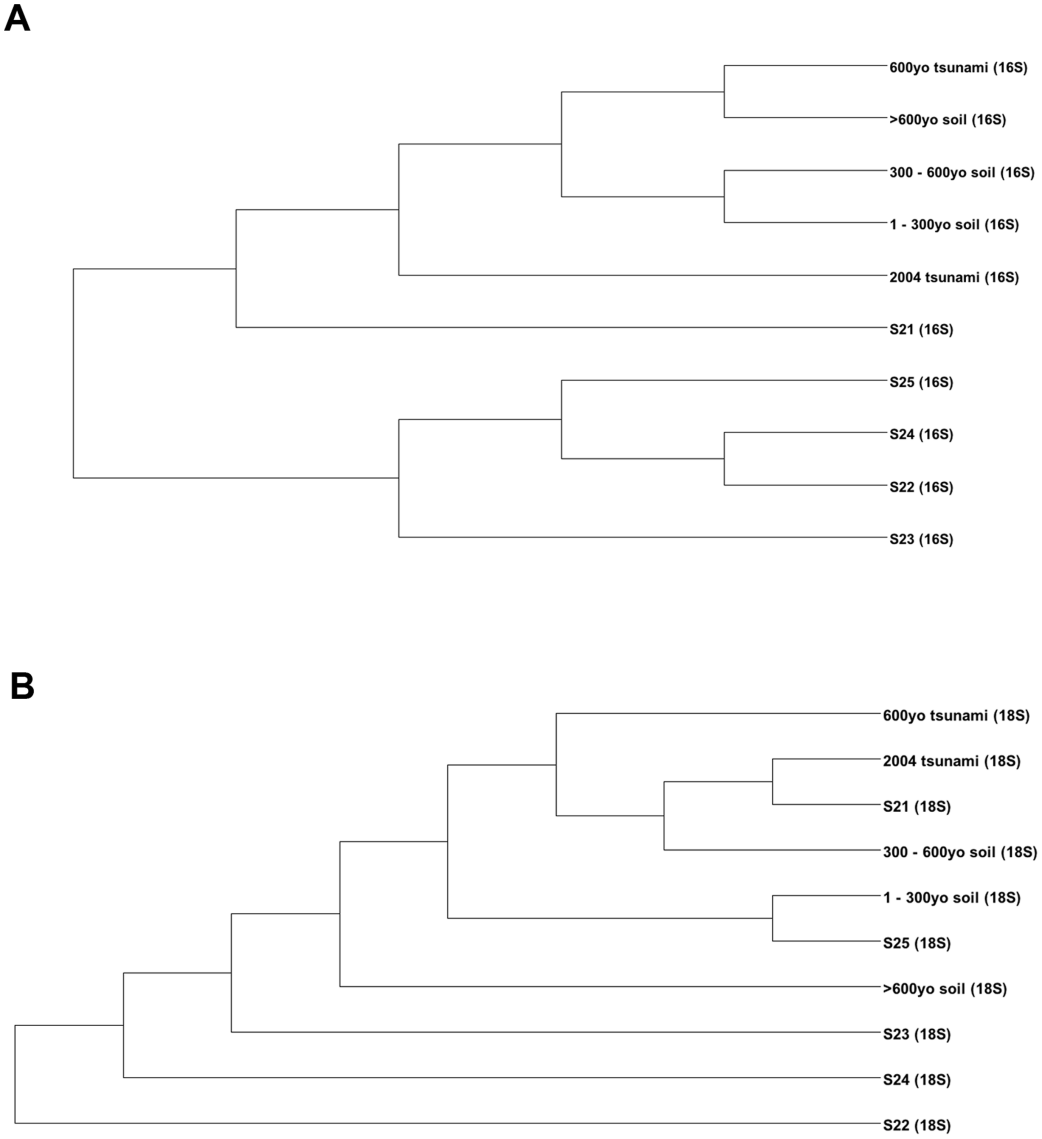
UPGMA clustering comparing relatedness among S_1_ and S_2_ prokaryotic (A) and eukaryotic (B) profiles.

Major prokaryotic phyla for S_1_ included Proteobacteria, Bacteroidetes, and Actinobacteria; and S_2_ included Proteobacteria, Acidobacteria, and Actinobacteria, in diminishing order (Figure 4A). Consistent with Figure 3, S_1_ demonstrated greater prokaryotic biodiversity than S_2_. S_1_ contained many new species belonging to uncultured species, i.e. OP3, GNO4 and SC3, whereas S_2_ still contained high proportion of common environmental phyla, including Proteobacteria and Acidobacteria (Figure 4A). Different sample periods showed slight variation of prokaryotic phyla profiles in S_1_, whereas in S_2_ more variation among the phyla distributions was evident (Figure 4B). For instances, Proteobacteria comprised 62.23% in the S_1_ layer of 2004 tsunami, 58.56% 1–300yo, 59.18% 300–600yo, 60.69% 600yo tsunami, and 62.54% >600yo; while S_2_ comprised 46.07% in the S21 layer, 65.72% S22, 77.64% S23, 75.05% S24, and 67.41% S25. Actinobacteria comprised 14.48% in S_1_ layer of 2004 tsunami, 13.22% 1–300yo, 13.53% 300–600yo, 13.82% 600yo tsunami, and 13.57% >600yo; while S_2_ layers demonstrated 14.32% S21, 7.59% S22, 6.52% S23, 8.92% S24, and 10.47% S25 (Figure 4B). Further distinguished differences were diagnosed upon analysis based on species distributions: no parallel-age pairs of S_1_ and S_2_ showed similar species distribution pattern with the >600 yo and S25 pair showing the least differences. Examples of predominated species in S_1_ were *Acinetobacter haemolyticus*, *Polynucleobacter* sp., *Polynucleobacter necessaries* and *Flavobacterium denitrificans*; and S_2_ were *Burkholderia* sp. and *Silvimonas terrae* (Figure 5). Subsequently, the computed Thetayc and Morisita-Horn dissimilarity indices revealed dissimilar community structures between S_1_ and S_2._ As shown in UPGMA dendrogram in Figure 6A: terrestrial layers corresponding to S_1_ site were clustered together separated from the S_2_ layers. Although S21 was grouped with the S_1_ layers, it was an outer related branch to the S_1_ group.

### Potential metabolic system analysis of prokaryotic communities

**Figure 7:**
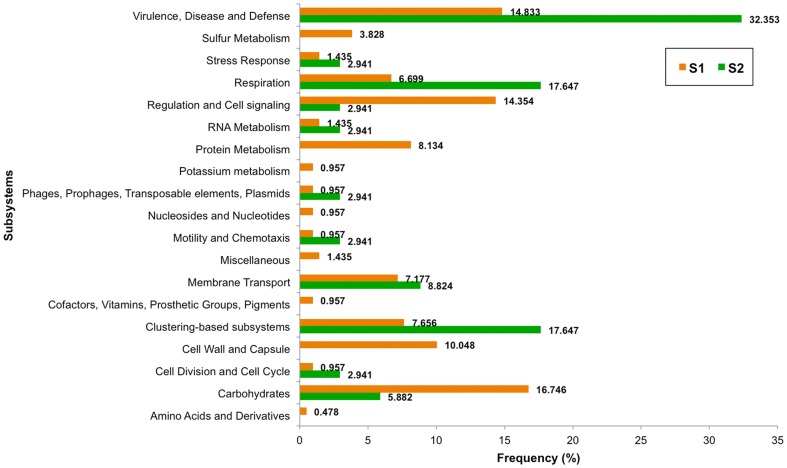
Metabolic subsystems of prokaryotic communities in S_1_ and S_2_.

From a total of 28 possible metabolic subsystems by mg-RAST [25], [26], [37], the prokaryotic communities of S_1_ contained 19 subsystems, and S_2_ contained 11 subsystems. The predominant subsystems in the S_1_ included: regulation and cell signalling (14.35%), cell wall and capsule (10.05%), protein metabolism (8.13%), and sulfur metabolism (3.83%) (Figure 7). Overall, S_1_ inhabited prokaryotic communities with high metabolic potentials for cell wall and capsule (i.e. gram-negative cell wall components by TIdE/PmbA protein), protein metabolism (i.e. protein degradation by prolyl endopeptidase), regulation and cell signaling (i.e. regulation of virulence by two-component system response regulator), and carbohydrates (i.e. sugar alcohols and central carbohydrate metabolism by HPr kinase/phosphorylase). S_2_ inhabited prokaryotic communities with high metabolic potentials for respiration (i.e. electron donating and accepting reactions by Type cbb3 cytochrome oxidase biogenesis protein), clustering based subsystems (i.e. copper-translocating P-type ATPase), and virulence, disease and defence (resistance to antibiotics and toxic compounds by acriflavin resistance protein).

### Diversity of eukaryotic phyla and species

**Figure 8:**
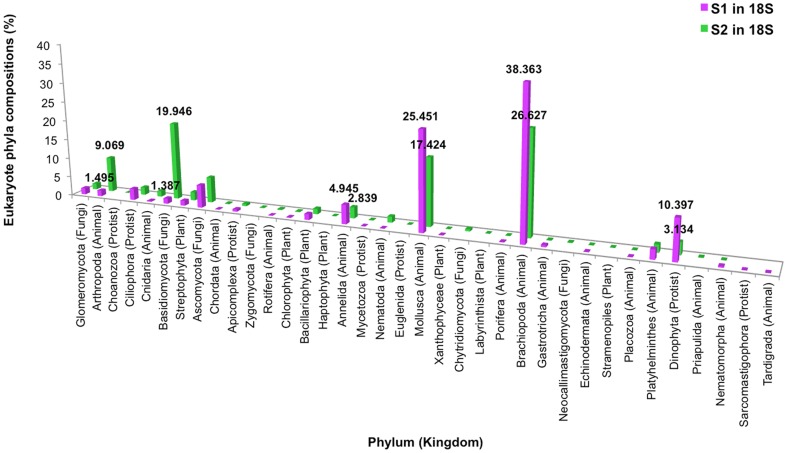
Distribution of eukaryotic phyla in S_1_ and S_2_, without (A) and with (B) individual sample ages categorization.

**Figure 9:**
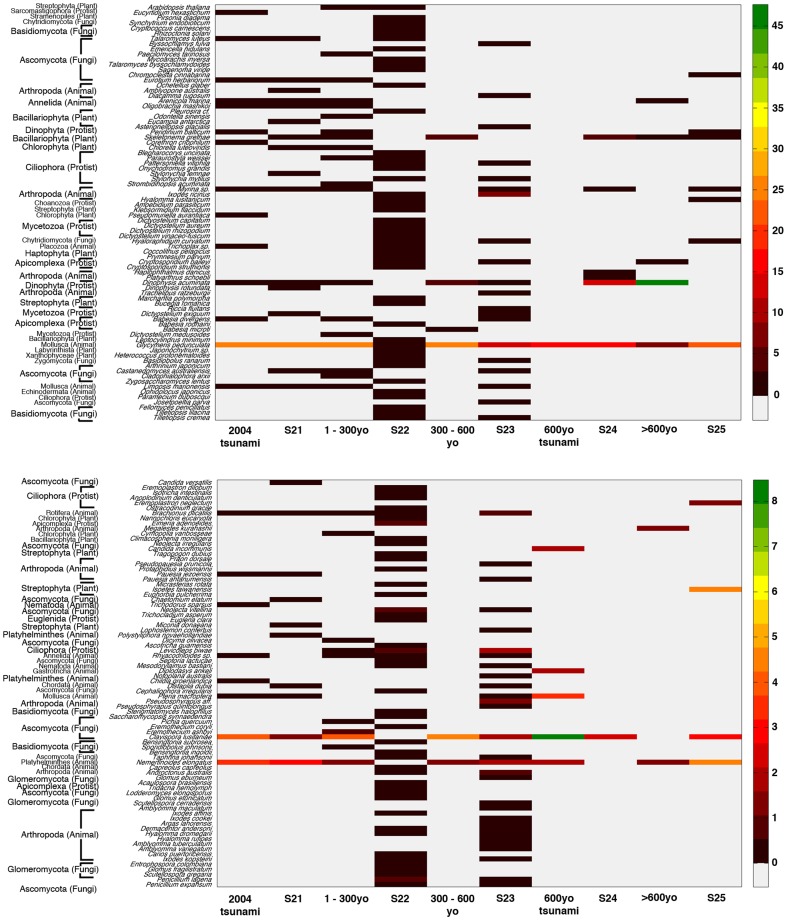
Distribution of eukaryotic species in S_1_ and S_2_, categorized by individual sample ages. Different color on the diagram represents a different relative abundance, based on the percent frequency chart on the right.

Dominant eukaryotic phyla for S_1_ were in kingdom Animalia: Brachiopoda (47.82% in 2004 tsunami, 32.53% 1–300yo, 37.32% 300–600yo, 49.15% 600yo tsunami, 25.00% >600yo), and Mollusca (28.88% 2004 tsunami, 29.39% 1–300yo, 31.34% 300–600yo, 30.51% 600yo tsunami, 7.14% >600yo); and kingdom Protozoa: Dinophyta for particularly the >600yo layer (51.99%) (Figures 8A and 8B). For S_2_, although Brachiopoda and Mollusca were dominant, fungal phylum Basidiomycota (0.08% S21, 66.51% S22, 7.47% S23, 12.82% S24, 12.86% S25) and animal phylum Arthropoda (3.05% S21, 3.42% S22, 28.33% S23, 7.70% S24, 2.86% S25) were the most prevalent. When analyzing the data into individual sample periods, similar finding to Figure 4B were found. Different sample periods of S_1_ demonstrated less phyla pattern variation than those of S_2_ (Figure 8B). Distinguished phyla pattern of S_2_ from S_1_ were displayed apparently in S22, S23 and S24 layers (Figure 8B), resulting in their divergence from S_1_ and the other S_2_ communities by the UPGMA dendrogram constructed using Thetayc dissimilarity indices (Figure 6B). Similar to prokaryotes (Figure 6A), the eukaryotic communities corresponding to the S_1_ site were relatively clustered together (Figure 6B). Analysis at the species level identified a more diverse fungal and animal species among S_2_ layers (Figure 9).

### Habitat classification

**Figure 10:**
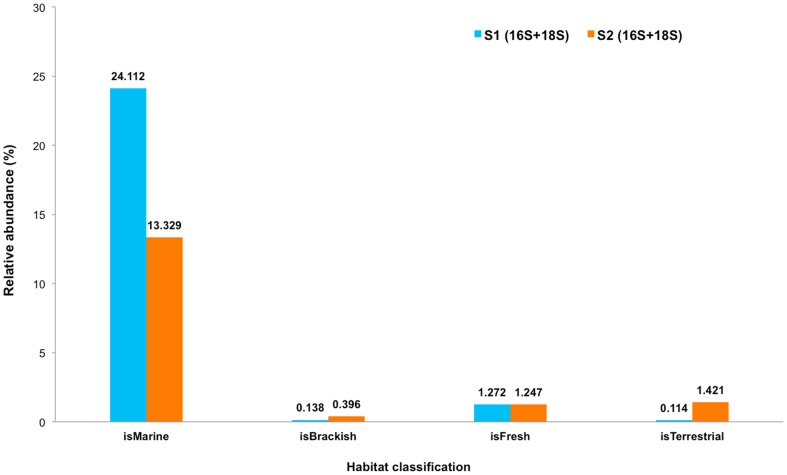
Habitat classification for S_1_ and S_2_. Prokaryotic and eukaryotic species profiles of S1 and S2 were matched against WoRMS database for habitat classification.

The S_1_ and S_2_ prokaryotic and eukaryotic profiles were matched against WoRMS database [38] to further characterize their microbial ecology: how each is related to marine, brackish water, freshwater, and terrestrial species communities. Figure 10 exhibited a substantially higher abundance of marine species habitat with S_1_ (S_1_ = 24.11%, S_2_ = 13.33%), and terrestrial species with S_2_ (S_1_ = 0.11%, S_2_ = 1.42%). Examples of abundant marine prokaryotes in S_1_ were: *Lutaonella thermophilus*, *Shewanella aquimarina*, *Erythrobacter ishigakiensis* and *Thalassobacter stenotrophicus*. Abundant marine eukaryotes in S_1_ included: *Dinophysis acuminata*, *Ctenodrilidae sp.*, *Remanella sp.*, *Nemertinoides elongatus*, *Skeletonema grethae*, *Crassostrea gigas*, *Hymenocotta mulli*, *Diplodasys ankeli*, *Pinna muricata*, *Arenicola marina*, *Limopsis marionensis* and *Haliplanella lucia*.

## Discussion

On-site records indicated the greater turbidity (Figure 1) and acidity of S_1_ were in agreement with previous reports [1], [2], [5]–[7]. Together with many other tsunami studies, these different soil types suggested terrestrial component changes following tsunami inundation, which could affect the microbial ecology of the site. The terrestrial microbiome representing the 2004 tsunami-affected site has never been studied. Our findings represent the first to utilize metagenomics in gaining databases of these entire terrestrial microbiomes, including prokaryotes and eukaryotes, in tsunami-affected (S_1_) and non-tsunami affected (S_2_) sites of Phra Thong island, as of March 2011. The data helped characterize the microbial biodiversity and its impact by tsunami occurrence. This knowledge is essential for scientists and engineers involved with land management and environmental bio-improvement.

Diminished total nucleic acids from S_1_ suggested a less populated microbial community. Although some fossil DNA and fragments of DNA from live animals (known as extracellular "dirt" DNA) could be included in the extracted metagenomes, and might partly complicate the analysis. Andersen et al. [39] found extracellular "dirt" DNA from the terrestrial surface could reflect an overall taxonomic richness and relative abundance of species of a site at the time of investigation. Hence, some extracellular "dirt" DNA in our extracted metagenome should also reflect an overall biodiversity.

Libraries of pyrotagged 16S and 18S rRNA gene fragments were successfully constructed and pyrosequenced: 21,592 reads for S_1_ and 33,308 reads for S_2_ were retrieved after removal of unreliable sequences. For BLASTN species identification, greater than 95% of the S_1_ and S_2_ reads were identified with ≤10^−5^ E-values. The amount of reads should be sufficient to recapture the relationships among the samples, as Caporaso et al. [40] reported 2,000 reads could recapture the same relationships among samples as did with the full dataset. Additionally, many studies discovered variable regions 3 and 4 of 16S rRNA gene analyses were more effective than random sequence reads analyses in estimating the biodiversity and relationships among the samples [41]–[43].

In S_1_, domain of prokaryotes became highly present (Figure 2). In particular, S_1_ had a richer archaeal population (4.07-fold increase), meanwhile fungi, animals, plants, and protists were decreased (Figure 3). This finding was consistent with the fact that tsunami inundation might leave a terrestrial site inhospitable, causing archaea and bacteria to be more common due to their flexible life activities and requirements [44]–[46]. The greater biodiversity of kingdoms in S_2_ supported the more hospitable terrestrial habitat than S_1_.

Phyla and species distribution patterns between S_1_ and S_2_ were different. Changing the prokaryotic pattern of major phyla, precisely S_1_ was predominantly comprised of Bacteroidetes with a lower prevalence of Proteobacteria, Actinobaceria and Acidobacteria (Figure 4A), highlighted the modified microbial ecology. Wada et al. [47] reported the similar change of bacterial floras in the sludge brought ashore by the 2011 East Japan earthquake. Bacteroidetes was more evident than Proteobacteria in the affected coastal water area. Additionally, numerous sulfate-reducing bacteria were evident in the sludge, which corresponded with high concentrations of sulfate ions in the sludge and the affected water area. The latter report was consistent with our finding of the higher sulfur metabolism in S_1_ (Figure 7). Further, among the Bacteroidetes, flavobacteria predominanted which is generally classified as an environmental bacterium with both commensal and pathogen species of marine animals and humans. Banning et al. [48] discovered several flavobacteria strains in various marine environments could function as predators on other bacteria. These bacteria have minimal growth requirements, only sea salt and the utilization of the lysed bacteria. The marine Flavobacteria thus could have critical consequences on microbial ecology as they could eradicate certain microbial communities [48]. Additionally, *Polynucleobacter* sp. are bacterioplankton that could survive broad ecological niches due to their ability to obtain energy by consuming organic materials from other organisms through nitrogen fixation, nitrification, remineralisation and methanogenesis. *Photobacterium* sp. is also a genus with metabolic versatility, which can degrade chitin and cellulose for carbohydrates [49]. Consequently, the flavobacteria, bacterioplankton and photobacteria activities could partly support the high metabolic subsystems of carbohydrates and protein metabolism in S_1_ (Figure 7), albeit the overall poor living condition. Note the increased regulation and cell signalling, and cell wall and capsule subsystems (Figure 7) could in part symbolize the growth activities of these bacteria, given capsule lies outside the bacterial cell wall and considered a virulent factor. Bacterial capsule protects the bacteria against some hostile environment, such as desiccation, and prevents phagocytosis by host immune cells [50]. For examples, *Acinetobacter haemolyticus* in contaminated seafood produces shiga toxin that causes bloody diarrhea [51], and *Flavobacterium* sp. cause cold water disease in salmon and other fish species [52]. *Photobacterium*, a genus in family *Vibrionaceae*, is primarily marine microorganisms that evolved to become pathogenic to marine animals, causing mortality in crabs and fish, and indirect pathogens of humans through contact or consumption [49], [53]. Hence residents and workers in these areas were recommended to minimize direct contact with the affected soil, sludge and water, to prevent their risk of infection, and frequent hand wash [3], [8], [9], [47]. Note the many new uncultured species in S_1_ (Figure 4A) further emphasized its environmental change resulting in new identified species. Our 16S rRNA gene analyses supported the high dissimilarity indices between S_1_ and S_2_ prokaryotic community structures (Figure 6A).

The metabolic potentials in Figure 7 supported the prior results, showing advanced metabolic subsystems of regulation and cell signalling, cell wall and capsule, protein metabolism, sulfur metabolism, and carbohydrates in S_1_. In contrast, S_2_ microbial communities carried high metabolic potentials for pathways of respiration, photosynthesis, and drug and bioactive compound production. This finding supported the diversified biodiversity in the non-affected terrestrials, and highlighted the more abundant pharmaceutical related microbial producers in the naturally undisturbed environments [15], like S_2_.

For eukaryotic phyla and species distribution patterns, while both mollusks and brachiopods predominated in both terrestrials, given both animals were marine animals and were more prominent in tsunami-inundated S_1_ site, fungi Basidiomycota and animal Arthropoda were only highly proportionate in the S_2_ area (Figure 8A). Like prokaryotic phyla distribution patterns among various terrestrial depths (Figure 4B), the more similar eukaryotic phyla distribution patterns among various terrestrial depths were evident in S_1_ (Figure 8B) highlighted the factor that a massive tsunami hit could destroy the biodiversity within microbial ecosystems. Basidiomycota, which were found more evident in S_2_, are higher fungi that play important roles as carbon recycler and nutrient decomposer, and posed the chief source of bioactive natural products [54]–[56].

Since the 2004 tsunami up to our study period, an effect of microbial population mixing and microbial change due to human or animal activity on S_1_ and S_2_ sites in Phra Thong island should be minimal. The reasons are because, after the 2004 tsunami, Phra Thong island remains almost no human inhabited and no human activity. The place becomes part of the wildlife sanctuary. Microbial population mixing through time could only be by rainfall and plant root penetration (mostly grass at both sites); hence it should be minimal and in vertical direction only.

Together, the 16S and 18S rRNA gene profiles indicated both terrestrials marine habitat, corresponding to the fact that the two sites were located on a small island in Andaman Sea of Thailand. Nevertheless, twice the greater prediction for marine habitat for S_1_ (Figure 10) highlighted its much higher number of prokaryotic and eukaryotic species that represent marine habitat species-indicators, and tsunami inundation.

## Conclusion

During the past decades, tsunamis have occurred more frequently though the correlation between tsunami disturbance and change of terrestrial microbial ecology remain poorly defined. The present study provided a culture-independent prokaryotic and eukaryotic analyses representing 0.22–30 μm metagenomes belonging to Thailand tsunami and non-tsunami affected terrestrials. The different prokaryotic and eukaryotic profiles highlighted the differences due to tsunami, and helped fulfill our knowledge of diverse terrestrial microbial ecologies. The biodiverse species of S_1_ distinguished its microbial communities and metabolic potentials. For instances, the finding of predator and prey bacterial relationship, and cell wall and capsule subsystem were common for S_1_, whereas bioactive compound producers were more common in S_2_.

Further, the marine habitat analysis demonstrating the greater percent of marine prokaryotic and eukaryotic species in S_1_ could perhaps help serve as another biomarker for the geological history of a terrestrial site. Nonetheless, more researches are required to utilize these as biomarkers for estimating times and presence of any geological incidences. Presently, identification of historic tsunami was restricted to examination of marine and brackish diatoms while silica shell of diatom could be dissoluted through time, especially in hot weather of a tropical country [1], [2], [57], [58].
